# Case Report: A patient with metastatic bladder cancer in the stomach

**DOI:** 10.3389/fonc.2025.1591475

**Published:** 2025-07-03

**Authors:** Shurong Liu, Junling Zhang, Xin Wang

**Affiliations:** Department of Gastrointestinal Surgery, Peking University First Hospital, Beijing, China

**Keywords:** metastatic tumor, bladder cancer, biopsy, digestive tract endoscopy, stomach tumor

## Abstract

Metastasis of bladder cancer to the stomach was rarely reported before. We reported a case of a 68-year-old woman with metastatic bladder cancer to the stomach. The patient was diagnosed with epithelial cell carcinoma of the bladder and underwent resection of the bladder, the right ureter and right kidney two years ago. One and a half years later, the enhanced CT examination revealed diffuse thickening of the stomach. Gastroscopy showed the gastric mucosa were swollen, congested and stiff. The biopsy of the EUS-FNA (Endoscopic Ultrasound-Guided Fine Needle Aspiration) showed a high likelihood of bladder epithelial tumors. Then the patient underwent a total gastrectomy and immunohistochemistry of the specimen indicated that her tumor tissue was originated from the bladder. We recommend that clinicians remain vigilant for gastric tumors that are metastases from bladder cancer, especially in patients with a previous primary bladder tumor.

## Introduction

Tumor metastasis to the stomach is rare. The reported incidence of gastric metastases is less than 2% (1). Most metastases to the stomach occur through the bloodstream route, such as malignant melanoma, breast and lung cancers. Metastasis occurs less frequently in thyroid, esophageal and kidney cancers (1–3). Reports of gastric metastases from urothelial carcinoma of the bladder are even extremely rare (4). Initially, Wallmeroth (5) and Pak (6) et al. separately identified 12 patients with gastric metastases in autopsy reports of patients with bladder cancer. However, none of these patients had imaging and follow-up records. In 2009, Hong et al. reported a case of metastatic gastric linitis plastica from bladder cancer mimicking a primary gastric carcinoma (7), imaging information is given for the first time. In 2022, a study on metastatic tumors in the stomach mentioned one case of metastatic tumor in the stomach originating from a high-grade sarcoma of the bladder (1). Overall, patients of gastric metastases from bladder cancer were very rare. Studies with well-established imaging data and surgical pathology were less. Our study reported a case of gastric metastasis from uroepithelial carcinoma of the bladder that underwent gastrectomy.

## Case report

A 68-year-old woman with previous history of hyperlipidemia had previously undergone partial thyroidectomy, cholecystectomy and sterilization. The patient had no previous family history of tumors or familial genetic disorders. She underwent TURBT (transurethral resection of bladder tumor), resection of the left kidney and ureter for urothelial carcinoma of the bladder 24 months ago. Postoperative pathology revealed urothelial carcinoma *in situ* was seen in the local mucosal overlying epitheliumand no metastatic cancer was seen in any of the surrounding lymph nodes. Immunohistochemistry result (**Supplementary Figure S1**): AE1/AE3+, CK8/18+, CK20+, CK7+, GATA3+, CK5/6+, Vim interstitial+, Ki67 localized high expression (40%). She then received 4 cycles of chemotherapy with the Gemcitabine and Carboplatin (**Supplementary Table S1**). Five months later, she underwent laparoscopic total resection of the bladder and uterus due to localized recurrence of the bladder cancer.

**Figure 1:**
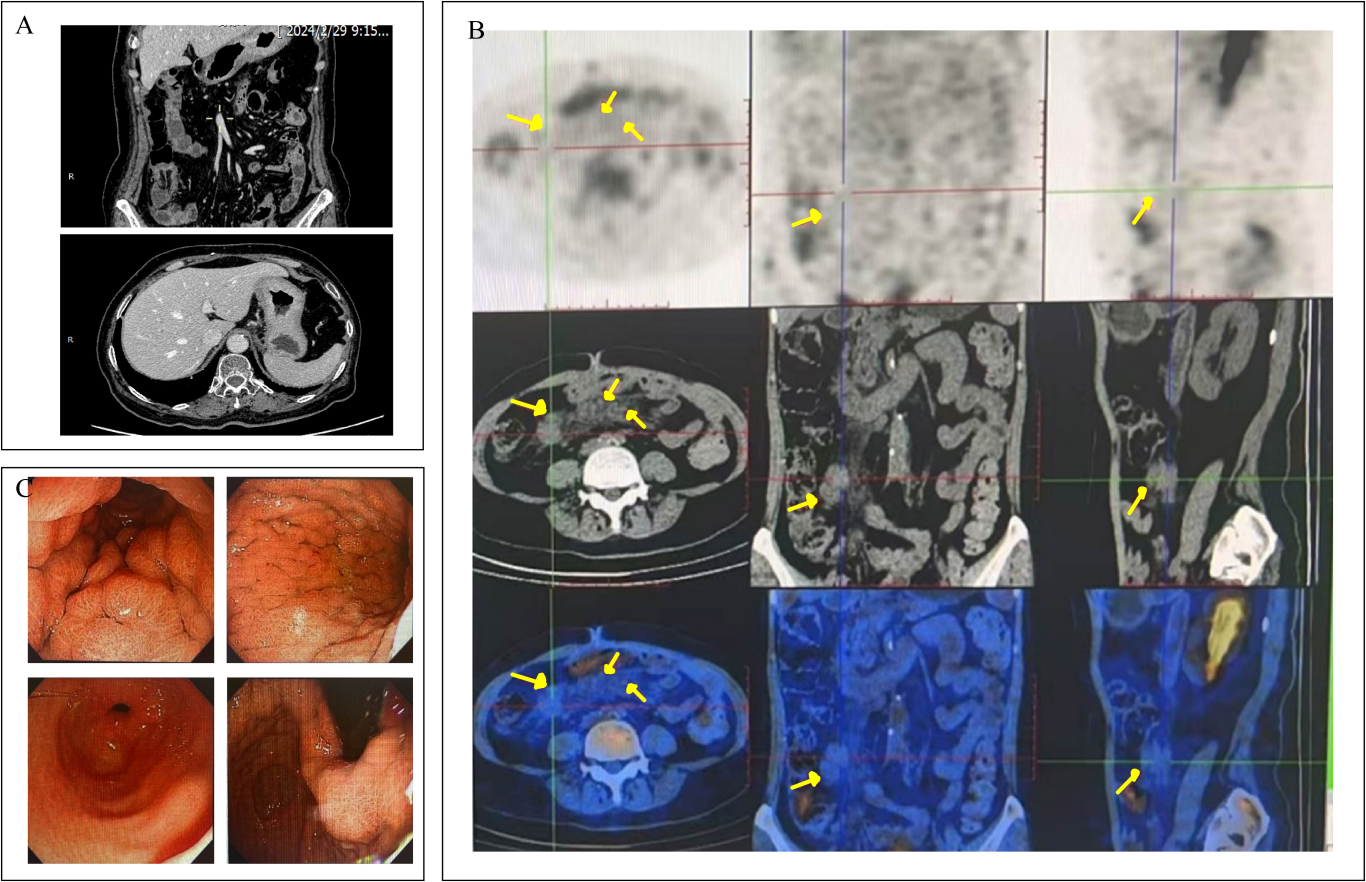
The CT, PET-CT and gastroscopic findings of the patient. **(A)** Contrast enhanced CT of the abdomen showed diffuse thickening of the gastric wall throughout the stomach. **(B)** PET-CT showed multiple hypermetabolic foci of mesenteric as well as retroperitoneal lymph nodes (locations marked with yellow arrows), which were considered to be metastases of the tumor. **(C)** Gastroscopy showed congested, edematous thickening of the gastric wall.

Sixteen months after surgery, the patient was readmitted to hospital for loss of appetite. Physical examination was unremarkable. The results showed that her hemoglobin concentration was 117g/L. Her tumor marker levels were up-regulated: CA19-9 >1000.0 U/ml, CA72–4 and CEA were within the reference range. Contrast-enhanced CT and PET-CT revealed diffuse thickening of the gastric body wall and the presence of multiple enlarged lymph nodes in the mesentery and retroperitoneum (**Figures 1A, B**). The swollen and congested gastric fundus mucosa was directly visualized by gastroscopy (**Figure 1C**). A small number of tumor cells were taken by EUS-FNA biopsy. Immunohistochemistry (IHC) showed that the tumor cells expressed a small amount of GATA3(+), which could be a tumor of bladder origin.

**Figure 2:**
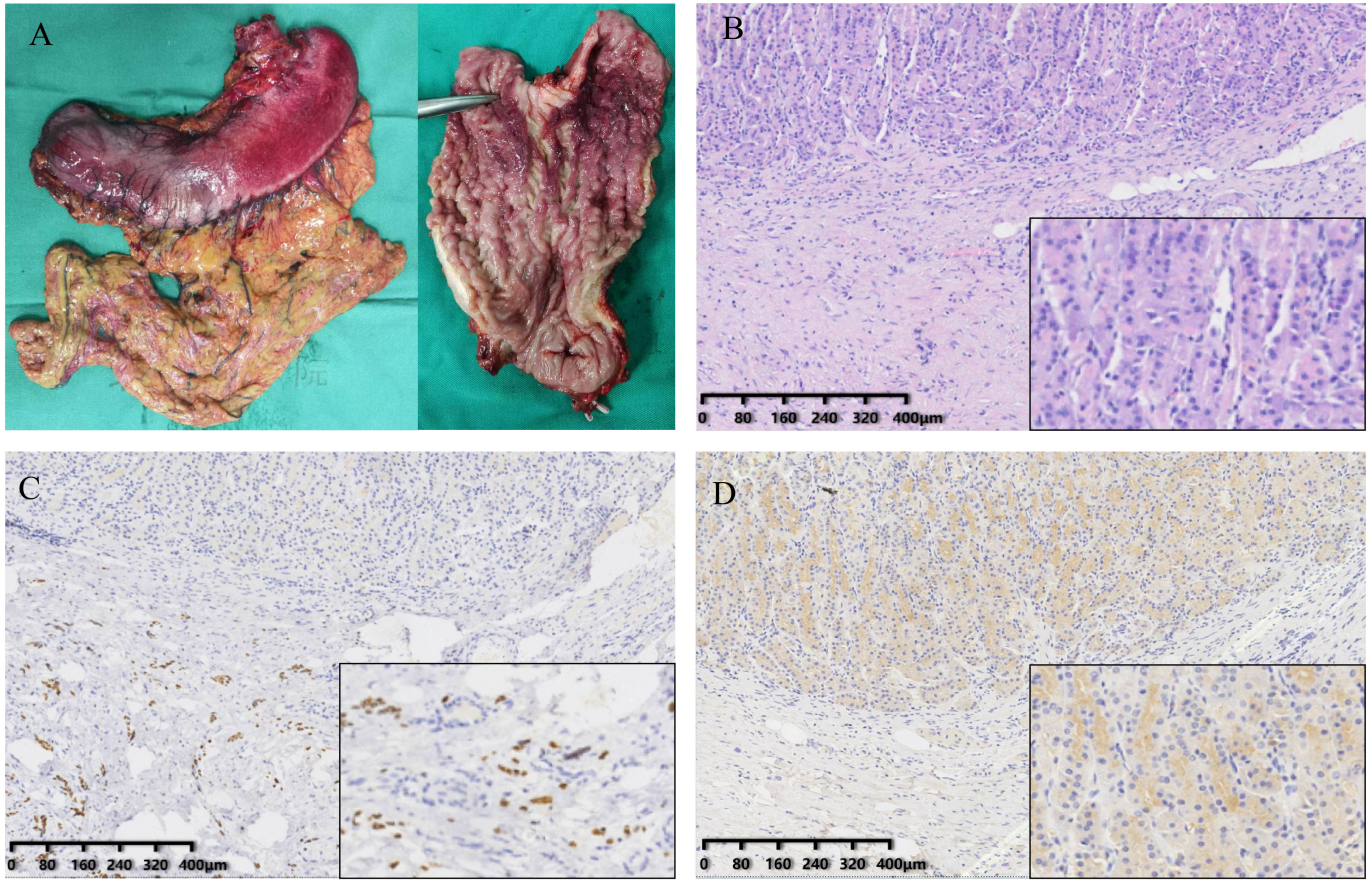
Gross specimens of the tumor and result of immunohistochemistry. **(A)** The stomach specimens. **(B)** The H&E staining of resected stomach. **(C)** GATA3 expression in the tissue. **(D)** CDX-2 expression in the tissue.

The patient then underwent palliative total gastrectomy. Lymph nodes no. 1, 3, 4, 5 and 6 were removed during the operation. The postoperative stomach specimen is shown in **Figure 2A**. Pathology suggested that the tumor was a poorly differentiated carcinoma (**Figure 2B**), with scattered tumor cells infiltrating the submucosa to the plasma membrane layer of the gastric wall. The tumor involved the mesothelium of the whole stomach and the dentate line, with nerve invasion but no vascular invasion. Cancer infiltration was present within the muscularis propria of the esophageal wall at the proximal margin (CK8/18+). No tumor cells were found in the distal pyloric margin (CK8/18-) and lymph nodes. Tumor cells were seen scattered within the fibro-fatty tissue in the lesser and greater curvature perimeter. IHC results: CK8/18+++, p53+++, Claudin18.2-, p40-, SATB2-, CK7+++, CK20-, CDX-2-, GATA3+++, Her-2 (0). Mismatch repair protein (pMMR): MLH1 (nuclear+), MSH2 (nuclear+), MSH6 (nuclear+), PMS2 (nuclear+). PD-L1: CPS is 0. ISH: EBV (EBER) negative. The results of IHC staining suggested strong positive staining for GATA3 (**Figure 2C**). CDX-2 results were negative (**Figure 2D**).

**Figure 3:**
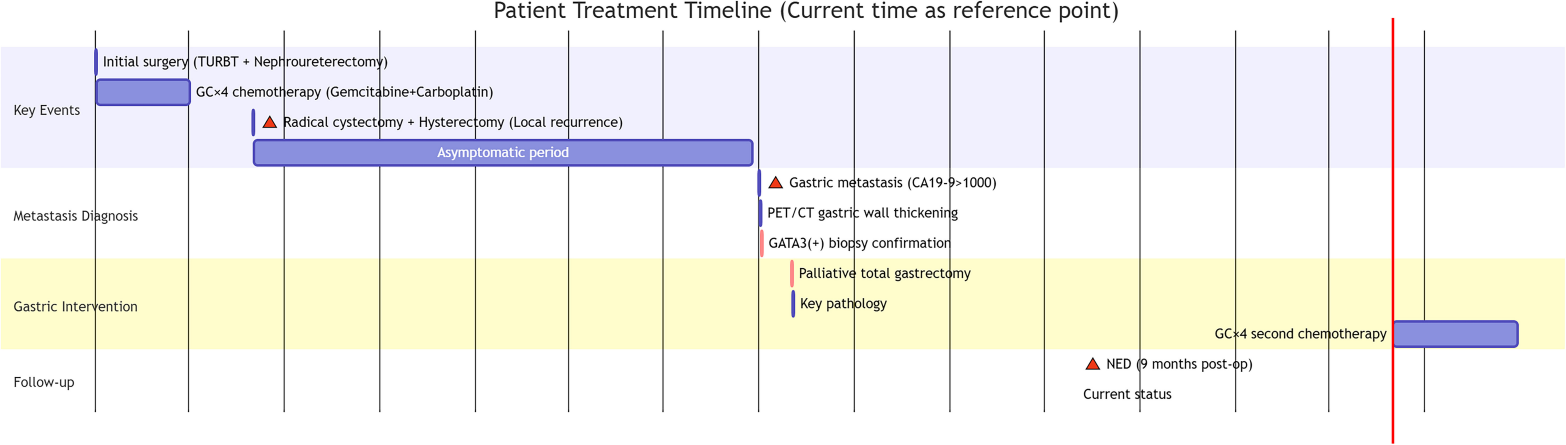
Timeline of clinical course and treatment interventions.

The patient’s pathology of two surgeries was similar (GATA3+), suggesting that the gastric tumor was a recurrence of uroepithelial carcinoma. The patient received 4 cycles of chemotherapy with the Gemcitabine and Carboplatin after the gastric surgery. Nine months after surgery, the patient had no evidence of recurrence and was leading a normal life. We provided a detailed treatment timeline of the patient’s visit (**Figure 3**).

## Discussion

Metastatic tumors of the stomach are very rare and the chance of developing is less than 2% (1). The most common types of tumor sources are breast cancer, melanoma, and lung cancer (2). In this context, the incidence of patients with stomach metastases from bladder cancer is even low. Despite the rarity of gastric metastasis, the stomach still needs to be considered as a target organ for metastatic tumors due to its abundant blood supply, especially in patients with a history of a primary tumor in other organs.

In recent years, only a small number of patients with gastric metastases from bladder cancer have been reported. In these patients who developed metastatic cancer of the stomach, the primary tumor type of the bladder is mostly uroepithelial cell carcinoma (7, 8), with only a few sarcomatoid lesions (1). Most patients present with metastatic lesions in the stomach found 2–5 years after the initial diagnosis of the primary tumor, and occasionally gastric metastases are seen as the first manifestation (9). These gastric metastases usually have a lesion pattern of single/multiple ulcers, polypoid masses or submucosal infiltration, which is highly susceptible to misdiagnosis as primary gastric cancer. For example, in the case reported by Hong et al (7), metastatic gastric cancer had features of the gastric mucosa that were similar to those of primary gastric cancer in terms of imaging presentation, showing a thickened gastric wall. Endoscopic biopsy pathology picked up only inflammatory cells. The gastric lesion was eventually clarified by surgical biopsy as a metastatic epithelial cell carcinoma of the bladder urothelium.

Metastatic gastric tumor can be a clinically fatal event, which implying poor prognostic survival for the patient (2). Therefore, it is important to determine whether a gastric tumor is primary or metastatic before implementing treatment programs. However, metastatic gastric tumors usually do not have specific clinical features. Patients may present with common gastrointestinal symptoms such as abdominal pain, anemia, and nausea. Some patients may not even have complaints of clinical discomfort, and only imaging data show abnormalities. It is difficult to distinguish whether a gastric tumor is primary or secondary singly on conventional contrast-enhanced CT alone. The use of new CT technology such as Spectral CT can be very helpful in making a diagnosis. A case of gastric metastasis from bladder cancer was reported recently (10), spectral CT and endoscopic biopsy were used to clarify that the gastric metastasis originated from bladder cancer. PET-CT allows a more comprehensive assessment of patient’s systemic metabolic status and hypermetabolic abnormalities at other sites, which can be helpful in determining the origin of the tumor. The better approach is gastroscopic EUS-FNA biopsy, which is extremely helpful in defining the pathology of gastric tumors by immunohistochemistry of the tissue type. The diagnosis of metastatic gastric cancer in our patient also benefited from the suggestion of preoperative EUS-FNA biopsy. However, endoscopic biopsies are limited in the number and location of biopsies taken, and false-negative results are also possible (7).

There are many challenges in the current research on gastric metastasis of bladder cancer. Firstly, patients face great difficulties in follow-up treatment. Bladder cancer is highly invasive, and traditional radiotherapy has limited therapeutic effects (11). For these patients with clear distant metastases, the value of surgical treatment is also limited, and is only applicable to some patients who are in good health condition and have no other distant metastases. The lack of a uniform treatment strategy for gastric bladder cancer metastases is obviously due to the small number of patients in previous studies. The patient in this case report underwent palliative gastric resection to alleviate clinical symptoms, and was treated with chemotherapy after surgery, and survived well at 9-month follow-up. Secondly, current research on the pathways by which bladder cancer develops gastric metastasis is unclear. The mainstream view is that bladder cancer breaks through the gastric mucosal barrier through the hematogenous metastatic route, however, without clear experimental evidence or related mechanism research. Lastly, the number of patient-reported cases is very small, with a very long period of time. The varying quality of imaging data and follow-up data in the reports make it difficult to conduct systematic studies.

We believe that future research should be directed toward the establishment of an international case registry to compensate for the lack of patients. A multidisciplinary treatment (MDT) is necessary to evaluate the characteristics of the disease in order to reduce the rate of misdiagnosis. More detailed history taking, as well as PET-CT and EUS-FNA biopsies are strongly recommend to identify those tumor tissues of suspicious origin to help surgeons make clinical decisions and provide them with individualized treatment options to improve the quality of patient survival. Finally, we have summarized an exhaustive treatment process (**Supplementary Figure S2**) to help maximize patient benefit.

## Data Availability

The original contributions presented in the study are included in the article/**Supplementary Material**. Further inquiries can be directed to the corresponding author.

## Supplementary material

Supplementary Table 1 Patient’s chemotherapy and remission status

Supplementary Figure 1 The result of immunohistochemistry. **(A)**. H&E staining of resected bladder cancer tissue. **(B)**. GATA3 expression in bladder cancer tissue. **(C)**. CK-7 expression in bladder cancer tissue.

Supplementary Figure 2 Clinical Decision Pathway for Suspected Gastric Metastasis from Urothelial Carcinoma.​​ This algorithm outlines the stepwise approach beginning with endoscopic biopsy and molecular testing for suspected metastatic disease. Confirmation of urothelial origin via immunohistochemistry is followed by metastatic staging with whole-body PET-CT. The therapeutic strategy is determined by the extent of metastasis: localized disease warrants surgical resection followed by adjuvant chemotherapy, while diffuse metastasis necessitates systemic therapy options (chemotherapy combined with immunotherapy or targeted agents).
