# Supplementary material for: Case Report: A patient with metastatic bladder cancer in the stomach
Source: Front Oncol. 2025 Jul 3;15:1591475. doi: 10.3389/fonc.2025.1591475 (PMC12267029; doi:10.3389/fonc.2025.1591475)
Supplement: Supplementary Table 1 — Patient’s chemotherapy and remission status [file Table1.docx]

**Table.S1 Patient's chemotherapy and remission status**

| **Time** | **BSA**  (m2) | **Treatment** | | **Adverse reactions** | **Assessment**  (Multi-stage enhanced CT) |
| --- | --- | --- | --- | --- | --- |
|  |  | **Gemcitabine**  (1000mg/m2) | **Carboplatin** (200mg/m2) |  |  |
| 1^st^ GC  (2022.6.28) | 1.50 | 1500mg | 300mg | No | Diffuse thickening of the bladder wall with multiple suspicious micronodular projections. There is localized thickening of the distal right ureter at its confluence with the bladder, and a slightly thicker wall in the middle part of the right ureter. Cysts or cystadenomas in both adnexal regions are possible. Dilatation of the right renal calyx, renal pelvis and ureter. |
| 2^nd^ GC  (2022.7.19) | 1.51 | 1500mg | 300mg | No | - |
| 3^rd^ GC  (2022.8.9) | 1.53 | 1500mg | 300mg | No | Diffuse thickening of the bladder wall was less than before. Micro-nodular protrusions of the bladder disappeared. Dilatation of the right renal calyx, renal pelvis and ureter is reduced. The right ureteral ratio thickening with hyperenhancement disappeared. |
| 4^th^ GC  (2022.8.30) | 1.51 | 1500mg | 300mg | No | Diffuse thickening of the bladder wall, as before. Slight thickening and roughness of the wall of the ureteral junction of the renal pelvis of the right kidney with high enhancement, newer than before, inflammation possible. |
