# Supplementary figures and images for: Case Report: A patient with metastatic bladder cancer in the stomach
Source: Front Oncol. 2025 Jul 3;15:1591475. doi: 10.3389/fonc.2025.1591475 (PMC12267029; doi:10.3389/fonc.2025.1591475)

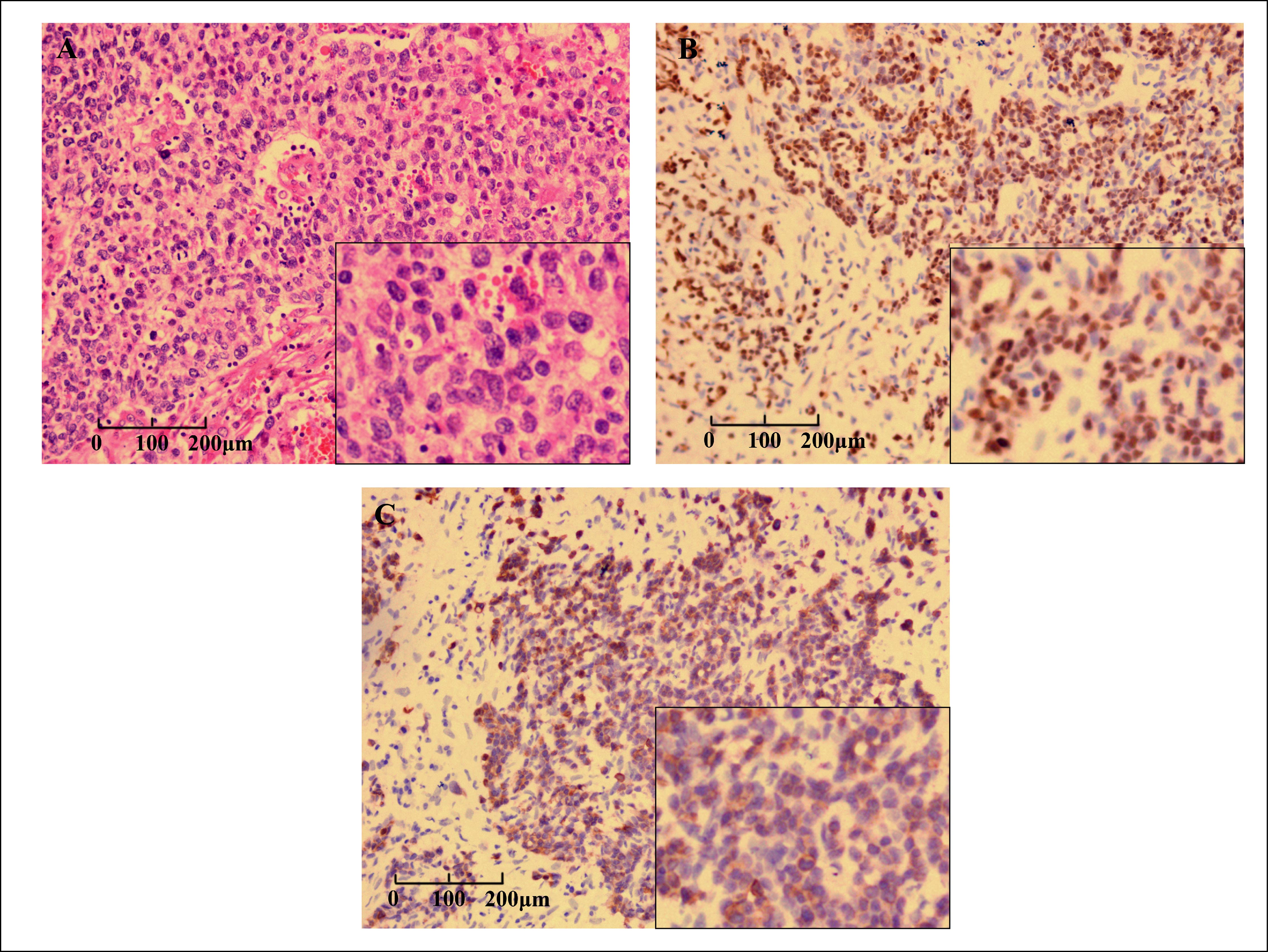

Supplement: Supplementary Figure 1 — The result of immunohistochemistry. (A). H&E staining of resected bladder cancer tissue. (B). GATA3 expression in bladder cancer tissue. (C). CK-7 expression in bladder cancer tissue. [file Image1.jpeg]

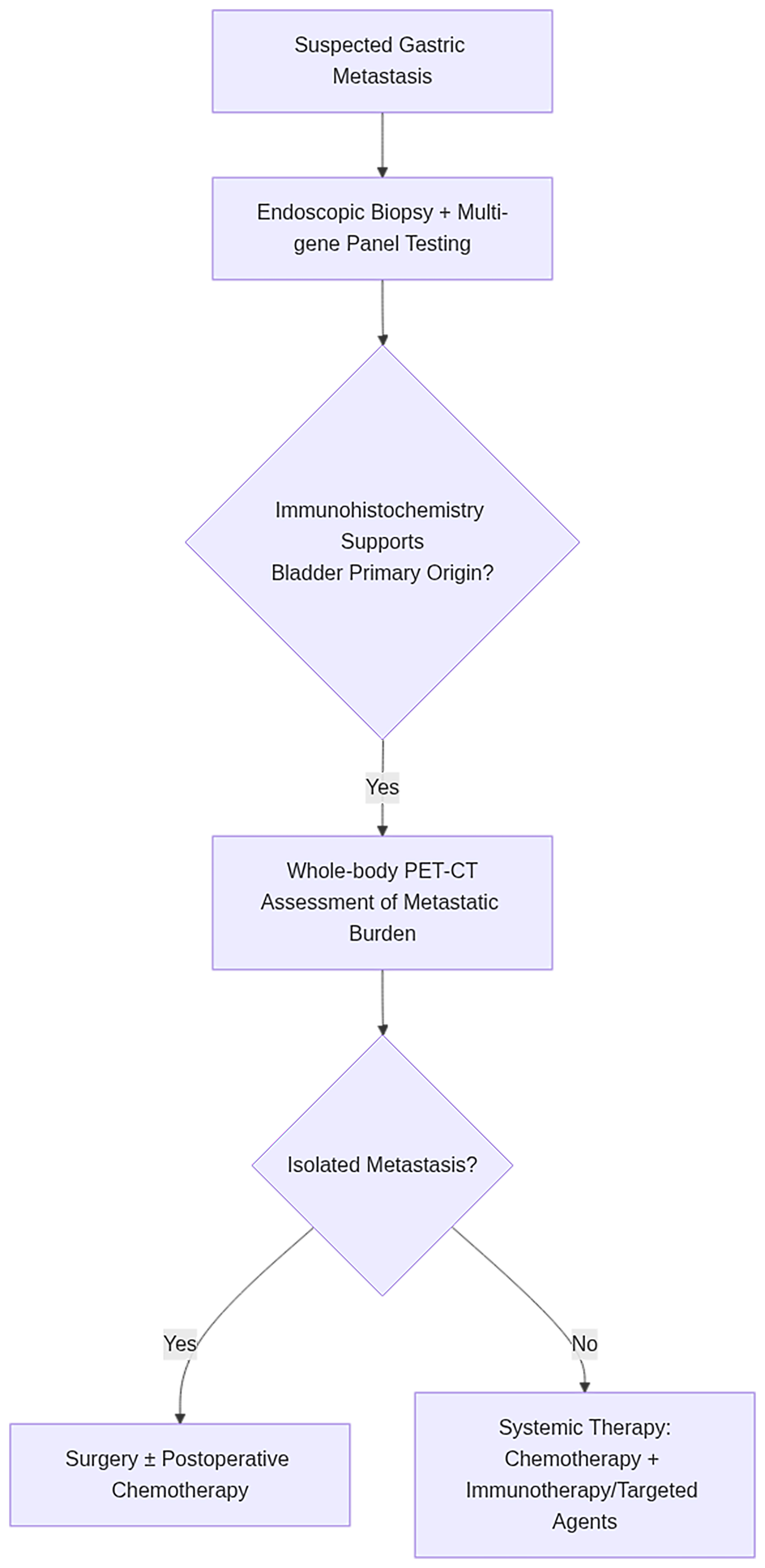

Supplement: Supplementary Figure 2 — Clinical Decision Pathway for Suspected Gastric Metastasis from Urothelial Carcinoma.​​ This algorithm outlines the stepwise approach beginning with endoscopic biopsy and molecular testing for suspected metastatic disease. Confirmation of urothelial origin via immunohistochemistry is followed by metastatic staging with whole-body PET-CT. The therapeutic strategy is determined by the extent of metastasis: localized disease warrants surgical resection followed by adjuvant chemotherapy, while diffuse metastasis necessitates systemic therapy options (chemotherapy combined with immunotherapy or targeted agents). [file Image2.jpeg]
